# Effects of cumulative trauma from multiple natural disasters

**DOI:** 10.1192/j.eurpsy.2023.2011

**Published:** 2023-07-19

**Authors:** B. Agyapong, R. Shalaby, E. Eboreime, G. Obuobi-Donkor, E. Owusu, M. Adu, W. Mao, F. Oluwasina, V. Agyapong

**Affiliations:** Department of Psychiatry, University of Alberta, Edmonton, Canada

## Abstract

**Introduction:**

Fort McMurray, a city in northern Alberta, Canada, has experienced multiple traumatic events in the last five years, including the 2016 wildfire, the 2020 floods, and the COVID-19 pandemic. Traumatic events often lead to increased mental health burdens in affected communities.

**Objectives:**

To assess if the number of traumatic events experienced by residents of Fort McMurray correlates with the prevalence and severity of mental health issues experienced.

**Methods:**

A cross-sectional study using an online survey questionnaire was used to gather demographic, trauma (wildfire, flooding, and COVID-19), and clinical information from the resident of Fort McMurray between April 24 to June 2 2021. Likely Generalized Anxiety Disorder (GAD), Major Depressive Disorder (MDD), Post-Traumatic Stress Disorder (PTSD) and low resilience were measured using standardized rating scales. Data were analyzed with SPSS version 26 using Chi-Square tests and multivariate regression analysis.

**Results:**

Respondents who experienced COVID-19 and either flood or wildfire traumas (*N* = 101) were eleven times more likely to have GAD symptoms (OR: 11.39; 95% CI: 1.43-91.04), four times more likely to have likely MDD, (OR: 3.85; 95% CI: .995-14.90), ten times more likely to have likely PTSD (OR: 10.47; 95% CI: 1.28-85.67), and low resilience (OR: 10.56; 95% CI: 1.21-92.17). Respondents who experienced COVID-19, flooding, and wildfire traumas (*N* = 47) were eighteen times more likely to express GAD symptoms (OR: 18.30; 95% CI: 2.20-152.45) and more than eleven times likely to have likely PTSD (OR: 11.41; 95% CI: 1.34-97.37) in comparison to the respondents who experienced COVID-19 only trauma (*N* = 19).

**Conclusions:**

Measures to reduce climate change and associated natural disasters could reduce the impact of cumulative trauma and associated mental health burden in vulnerable populations. It is essential that more mental health resources are mobilized to support communities impacted by multiple natural disasters.

**Disclosure of Interest:**

None Declared

